# Clinical Outcomes of Third-Generation Cephalosporin Definitive Therapy for Bloodstream Infections Due to Enterobacterales with Potential AmpC Induction: A Single-Center Retrospective Study

**DOI:** 10.3390/pathogens12091152

**Published:** 2023-09-11

**Authors:** Gilles Vossius, Cécile Meex, Filip Moerman, Marie Thys, Marie Ernst, Marie-Eve Bourgeois, Léa Wagner, Thibaut Delahaye, Gilles Darcis

**Affiliations:** 1 Département des Maladies Infectieuses, Centre Hospitalier Universitaire de Liège, 4000 Liège, Belgium; 2 Service de Microbiologie Clinique, Université de Liège, 4000 Liège, Belgium; 3 Département des Maladies Infectieuses, Hôpital de la Citadelle, 4000 Liège, Belgium; 4 Service des Informations Médico-Économiques, Centre Hospitalier Universitaire de Liège, 4000 Liège, Belgium; 5 Biostatistics and Research Method Center (B-STAT), Centre Hospitalier Universitaire de Liège, 4000 Liège, Belgium; 6 Faculté de Médecine, Université de Liège, 4000 Liège, Belgium

**Keywords:** AmpC, Enterobacterales, Enterobacteriaceae, beta-lactamases, cephalosporins, antimicrobial resistance

## Abstract

The recommended therapy for severe infections caused by AmpC-inducible Enterobacterales (AmpC-E) typically involves cefepime or carbapenems. In an era of emerging resistance to these antimicrobials, we aim to assess the impact of third-generation cephalosporins (3GCs) vs. alternative antibiotics on clinical outcomes in bloodstream infections (BSIs) due to AmpC-E. We retrospectively included hospitalized adult patients with BSIs caused by 3GC-susceptible AmpC-E between 2012 and 2022, comparing the outcomes of 3GC and non-3GC definitive therapies. The primary outcome was overall treatment failure (OTF), encompassing 90-day all-cause mortality, 90-day reinfection, and 90-day readmission. Secondary outcomes comprised components of the OTF, in-hospital all-cause mortality, and length-of-stay. Within a total cohort of 353 patients, OTF occurred in 46.5% and 41.5% in the 3GC- and non-3GC-therapy groups, respectively (*p* = 0.36). The 3GC-therapy group exhibited a longer length-of-stay (38 vs. 21 days, *p* = 0.0003) and higher in-hospital mortality (23.3% vs. 13.4%, *p* = 0.019). However, the 90-day mortality, 90-day reinfection, and 90-day readmission were comparable between the therapy groups. Subgroup analyses involving high-risk AmpC-E and 3GC vs. standard-of-care yielded similar conclusions. Overall, our findings suggest that 3GC definitive therapy may not result in poorer clinical outcomes for the treatment of BSIs caused by AmpC-E.

## 1. Introduction

Antimicrobial resistance in Gram-negative bacteria is an increasingly concerning global issue [1]. The production of β-lactamase enzymes is the primary mechanism driving this resistance [2]. The Centers for Disease Control and Prevention (CDC) and the World Health Organization (WHO) have both characterized β-lactamase-producing Gram-negative bacteria as one of the most serious medical threats worldwide [3,4].

Among these Gram-negative bacteria, certain Enterobacterales can harbor chromosomally encoded inducible AmpC-type β-lactamase enzymes, referred to as AmpC-producing Enterobacterales (AmpC-E). These organisms include the *Enterobacter cloacae* complex, *Klebsiella aerogenes* (formerly *Enterobacter aerogenes*), *Citrobacter freundii, Serratia marcescens*, *Providencia stuartii*, *Morganella morganii*, and *Hafnia alvei*. AmpC expression is constitutively weak. AmpC hydrolyzes penicillin G, penicillin A, and first-generation cephalosporins at a low level of expression, and piperacillin, second-generation cephalosporins, third-generation cephalosporins (abbreviated as 3GCs), and aztreonam at a high level of expression. The hydrolysis rates for fourth-generation cephalosporins and carbapenems remain very low [5].

One common therapeutic approach for severe infections, such as bloodstream infections (BSIs), caused by Enterobacterales is the use of 3GCs. However, there are concerns that exposure to certain antimicrobials, including 3GCs, may select for Enterobacterales overproducing AmpC, leading to initial in vitro susceptibility but potential resistance emergence and treatment failure in vivo. Additionally, AmpC expression varies among species, with higher levels observed in the *E. cloacae* complex, *K. aerogenes*, *C. freundii*, and *H. alvei* [6]. Currently, there is no method endorsed by the Clinical and Laboratory Standards Institute (CLSI) or the European Committee on Antimicrobial Susceptibility Testing (EUCAST) for detecting nonacquired AmpC β-lactamases, and the routine availability of AmpC testing in laboratories is limited [7,8]. Therein lies the therapeutic dilemma: should an AmpC-E infection with in vitro 3GC-susceptibility be treated with 3GCs, or does the risk of AmpC-mediated resistance emergence outweigh the benefits?

Guidelines generally advise against using 3GC therapy for AmpC-E infections based on the literature predominantly focusing on microbiological outcomes [9]. However, there is significant heterogeneity among studies, with varying rates of resistance emergence [10,11,12,13,14,15]. In contrast to these findings, recent studies suggest comparable clinical outcomes between 3GCs and other antibiotics for AmpC-E infections, although data on this topic are limited [16,17,18]. Therefore, the objective of this study is to determine whether the use of 3GC definitive therapy for BSIs caused by AmpC-E results in inferior clinical outcomes compared to non-3GC definitive therapy.

## 2. Materials and Methods

### 2.1. Design and Population

This study follows a retrospective, observational design conducted at the University Hospital of Liège, Belgium. Approval for the study protocol was obtained from the local ethics review committee (Comité d’Ethique Hospitalo-Facultaire Universitaire de Liège, reference number 2023-8, 14 February 2023). We included adult patients (≥18 years) who were hospitalized and presented with BSIs caused by AmpC-E that demonstrated in vitro susceptibility to cefotaxime and ceftazidime. Within our institution, the susceptibility of AmpC-E strains to 3GCs varies between 70% and 100%, depending on the pathogen under consideration. The inclusion period spanned from 1 January 2012 to 31 December 2014, and from 1 January 2016 to 30 September 2022. Patients with AmpC-E BSIs occurring in 2015 were excluded due to limitations in the medical filing system hindering data extraction during that period. Identification of eligible patients was conducted using the microbiology laboratory database. For patients with multiple episodes of AmpC-E BSI, only the first episode fulfilling inclusion criteria was analyzed. Exclusion criteria included the absence of antimicrobial therapy within three days of the index blood culture, death within three days of the index blood culture, discontinuation of antimicrobial therapy before five days of treatment, combination therapy with more than one drug providing anti-Gram-negative coverage (with the exception of aminoglycosides), missing data impeding the determination of treatment or outcomes, and polymicrobial BSIs based on the index blood culture. The determination of potential contaminants in the index blood culture was made by reviewing each patient’s medical file with an infectious diseases specialist.

### 2.2. Definitions

AmpC-E BSI was defined by at least one positive blood culture for the *E. cloacae* complex, *K. aerogenes*, *C. freundii*, *P. stuartii*, *S. marcescens*, *M. morganii*, or *H. alvei*, accompanied by symptoms and signs of infection. Empiric therapy was defined as any antibiotic administered within 72 h of the index blood culture and preceding definitive therapy. Definitive therapy was defined as the antibiotic chosen by the clinician after determination of antibiotic susceptibility, according to drug administration records and documentation in the daily rounds file. Definitive therapy could be categorized as “3GC” (i.e., cefotaxime, ceftriaxone, and ceftazidime) or “non-3GC” (i.e., any antibiotic except these). Adequate therapy was determined as any antibiotic for which the BSI pathogen was deemed susceptible in vitro based on the EUCAST interpretive criteria. Intravenous and oral administration routes were not differentiated for drugs with high oral bioavailability, such as fluoroquinolones, trimethoprim-sulfamethoxazole (TMP-SMX), and minocycline.

The primary composite outcome, overall treatment failure (OTF), encompassed three components: death from any cause within 90 days of the index blood culture, reinfection with the same organism within 90 days of the index blood culture (regardless of the source of infection), and readmission for nonelective reasons within 90 days of hospital discharge. Reinfection was assessed by comparing the drug-susceptibility profiles of the organisms and evaluating the clinical picture through medical records to differentiate between colonization and genuine reinfection. This definition of reinfection has limitations arising from the retrospective nature of our study, which precluded genotypic testing. Consequently, it is conceivable that patients may have acquired a new infection caused by a different strain of the same pathogen with the same susceptibility profile rather than experiencing a relapse with the same strain. Readmission was deemed nonelective if the admission grounds were acute and unforeseen (i.e., hospitalization for dialysis, chemotherapy without complication, and removal of central venous catheter were considered elective). The distinction between elective and nonelective readmission was determined by reviewing the reasons and conclusions of each hospital stay.

### 2.3. Outcomes

The primary outcome was overall treatment failure. Secondary outcomes included the individual components of the OTF, in-hospital all-cause mortality, and hospital length-of-stay. The principal analysis compared 3GC definitive therapy to non-3GC definitive therapy for patients presenting BSIs due to any AmpC-E. The first subgroup analysis analyzed 3GC definitive therapy vs. the current standard-of-care (i.e., cefepime or carbapenems) for patients presenting BSIs due to any AmpC-E. The second subgroup analysis evaluated 3GC vs. non-3GC definitive therapy for patients presenting BSIs exclusively due to high-risk AmpC-E (i.e., the *E. cloacae* complex, *K. aerogenes*, and *C. freundii*).

### 2.4. Data Management

Information was extracted from the medical files by the local data-analysis team. Basic parameters, such as age, gender, weight, and height, were recorded by the nursing team in a standardized format. Penicillin allergy was determined based on patient testimony obtained during anesthesiology consultations or encoded by the administrative team using International Classification of Diseases (ICD) codes. Intensive care unit (ICU) admission and the length of stay were retrieved from the admissions department records. Glomerular filtration rate was approximated by calculating the median value from all recorded measurements throughout the hospital stay.

Determination of comorbidities to compute the Charlson Comorbidity Index was based on ICD-9 and ICD-10 codes, as proposed by Quan et al. in 2005 [19]. This approach was chosen for its ease of data extraction and its ability to assess in-hospital mortality and resource utilization. To ensure comparability between ICD-9 (used before 2015 in our institution) and ICD-10 (used after 2015), we compared the frequency of each component in our study population using both codebooks and found no statistically significant difference (Supplementary Table S1).

Components of the qPitt and qSOFA scores were obtained through different methods. Systolic blood pressure and body temperature were extracted from the standardized nursing records. Respiratory rate and the presence of altered mental status were determined through a review of the daily rounds records. Occurrence of cardiac arrest was based on the appropriate ICD code and review of the daily rounds records. These parameters were considered positive if they occurred within two days before or after the index blood culture. Source of BSI was determined by the attending clinician and extracted through a comprehensive review of each patient’s medical file.

Empiric and definitive therapy, as well as the use of aminoglycosides, were determined by conducting a detailed review of the hospital pharmacy records and each patient’s medical file by an infectious diseases specialist. Dates of death were extracted from the national registry, with the last check performed on 31 January 2023.

### 2.5. Statistical Analysis

Categorical variables are expressed as numbers (%) and compared with a Chi-square test (or exact Fisher test if the sample size is too small). Quantitative variables are reported as mean and standard deviation (SD) or median and interquartile range (Q1; Q3), and compared using the Student’s t test (or Kruskal–Wallis test if a nonparametric test is required).

Kaplan–Meier plots were used to describe survival-type variables. Simple and multiple Cox regression models were used to assess the effects of 3GC therapy and other factors on survival, readmission, and reinfection. In multiple analysis, an adjustment was made for severity scores (qPitt and qSOFA) and ICU admission. Logistic regression models were performed to determine the impact of 3GC therapy on binary outcomes (OTF and in-hospital all-cause mortality). Two additional subgroup analyses were performed to investigate the differences for 3GCs vs. cefepime/carbapenems for all AmpC-E and 3GCs vs. non-3GCs only for the high-risk AmpC-E. Due to the fact that severity scores are correlated between them, a backward selection was performed to each model of the main and subgroup analysis, and the most appropriate severity score was kept. Detailed simple and multiple analysis are presented in the supplementary materials (Supplementary Tables S4, S6 and S8).

Post hoc power calculation, taking into account our sample size (*n* = 353), estimated hazard ratios derived from Cox regression models, a significance level of α = 0.05, and event rates spanning from 5.4% to 25.7% for different outcomes encompassing both primary and secondary outcomes, has provided us with the assurance that our attained statistical power exceeds 99%.

A *p*-value < 0.05 was considered significant and Bonferroni correction was used to deal with multiple testing (*p*-values were compared to 0.05 divided by the number of simultaneous tests). Missing data were not replaced. Calculations were performed using SAS (Version 9.4; Analytics Software and Solution, SAS Institute, Cary, NC, USA) and R (Version 4.2.2; R Foundation for Statistical Computing, Vienna, Austria).

## 3. Results

### 3.1. Participants

**Figure 1:**
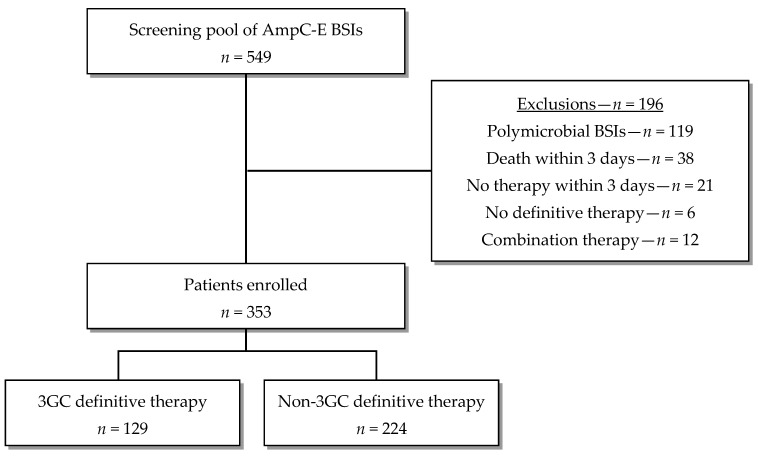
Flow diagram of patient enrollment.

A total of 549 patients with 3GC-susceptible AmpC-E BSI underwent screening for inclusion. Among them, 196 patients were excluded primarily due to the presence of polymicrobial index blood culture, predominantly associated with intra-abdominal and catheter-related infections. Ultimately, 353 patients were enrolled in the study, with 129 receiving the 3GC definitive therapy and 224 receiving the non-3GC definitive therapy (Figure 1).

The study population was similar between the 3GC and non-3GC groups as far as basic characteristics (age, gender, BMI, renal function, and penicillin allergy), adequacy of antimicrobial therapy, aminoglycoside use, and source of infection were concerned (Table 1). The Charlson comorbidity index was comparable in both groups (median 4.2 vs. 4.1, *p* = 0.70) (Supplementary Table S2). However, the 3GC group displayed significantly higher illness severity (ICU admissions 41.9% vs. 25.4%, *p* = 0.0014; mean qPitt score 1.55 vs. 1.13, *p* = 0.0017; mean qSOFA score 1.22 vs. 0.86, *p* = 0.0017). The difference in severity scores was mainly attributed to a higher frequency of respiratory distress (28% vs. 13%) and mental-status alteration (40% vs. 21%) in the 3GC-definitive-therapy group (Supplementary Table S3).

The most prevalent organism identified was the *E. cloacae* complex (41.6%), followed by *S. marcescens* (28%), *M. morganii* (12.5%), *K. aerogenes* (9.3%), and *C. freundii* (7.4%). *P. stuartii* was isolated in only three patients (0.8%) and *H. alvei* in one patient (0.3%). The distribution of these organisms was comparable between therapy groups (Table 1).

### 3.2. Antibiotic Therapy

Antibiotic usage was evaluated for all 353 patients (Table 2). Empiric therapy consisted of piperacillin–tazobactam for 135 patients (38.2%), 3GCs for 103 patients (29.7%), meropenem for 31 patients (8.8%), and ciprofloxacin for 25 patients (7.1%, primarily upper urinary tract infections). The 3GC definitive therapy involved cefotaxime in most cases (44.2%), followed by ceftriaxone (34.1%) and ceftazidime (21.7%). Non-3GC definitive therapy was split between β-lactams with a broader spectrum than 3GCs, such as piperacillin–tazobactam (25%), cefepime (8%), and carbapenems (8.5%), and step-down therapy with high oral bioavailability, such as quinolones (38.8%) and TMP-SMX (15.6%).

### 3.3. Outcomes for Principal Analysis—3GCs vs. Non-3GCs for All Amp-C E

**Figure 2:**
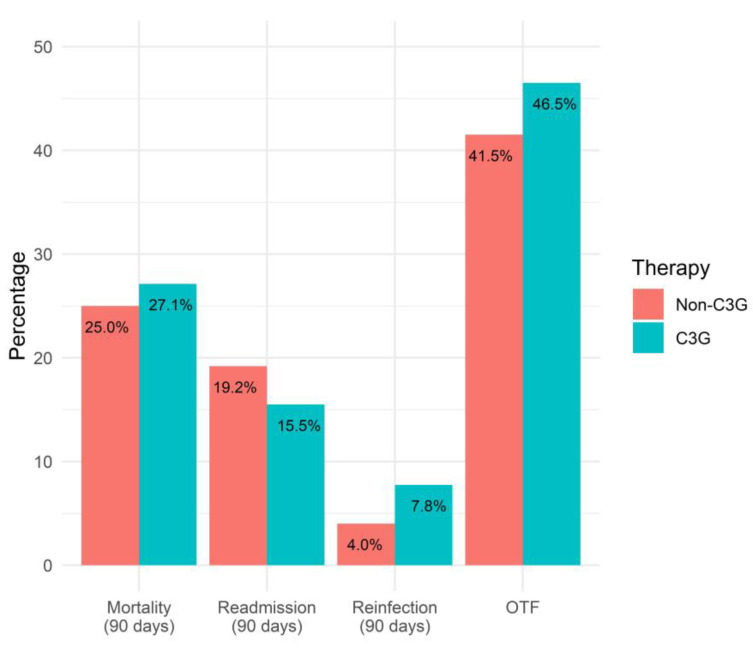
Comparison of OTF and its components for 3GC- and non-3GC-therapy groups for patients presenting BSIs due to any AmpC-E.

The primary outcome of the OTF was comparable between the definitive-3GC-therapy and non-3GC-definitive-therapy groups (46.5% vs. 41.5%, *p* = 0.36) (Figure 2). The logistic regression model showed similar odds of the OTF with the 3GC definitive therapy compared to the non-3GC definitive therapy (OR 1.22, 95%CI 0.79–1.90). Secondary outcomes included each component of the OTF, length-of-stay, and in-hospital mortality (Table 3).

**Figure 3:**
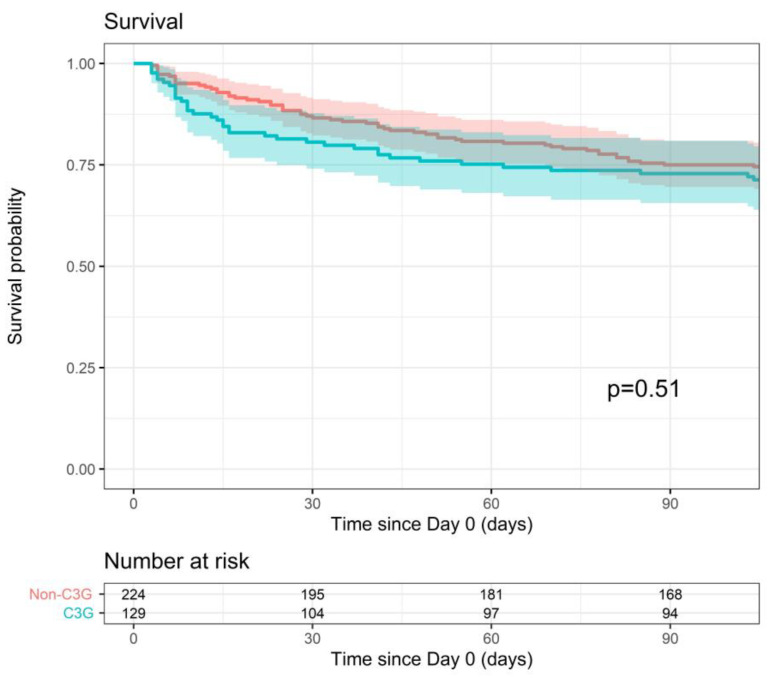
Survival within 90 days after index blood culture for 3GC- and non-3GC-therapy groups for patients presenting BSIs due to any AmpC-E.

All-cause mortality within 90 days of the index blood culture was comparable for the 3GC- and non-3GC-definitive-therapy groups (90-day survival probability of 72.9% vs. 75%, *p* = 0.51) (Figure 3). This outcome was significantly associated in the multiple analysis with the qPitt score. However, the adjusted model did not alter the conclusion (*p* = 0.54).

**Figure 4:**
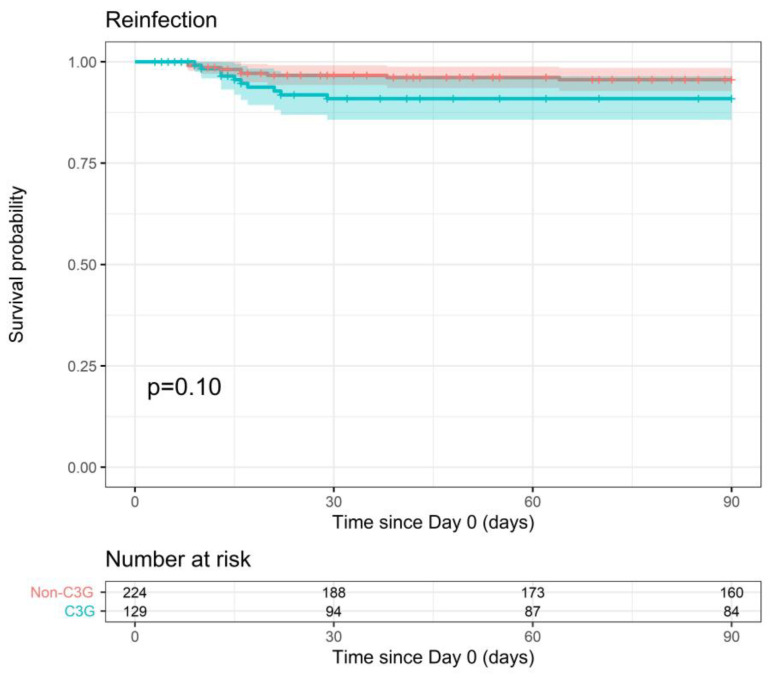
Probability of reinfection within 90 days after index blood culture for 3GC- and non-3GC-therapy groups for patients presenting BSIs due to any AmpC-E.

Reinfection with the same pathogen within 90 days of the index blood culture was similar for both therapy groups (90-day reinfection probability of 9.1% vs. 4.4%, *p* = 0.10) (Figure 4). This outcome was slightly associated with ICU admission in the multiple analysis (*p*-value < 0.1), but the adjusted model did not change the conclusion either (*p* = 0.19).

**Figure 5:**
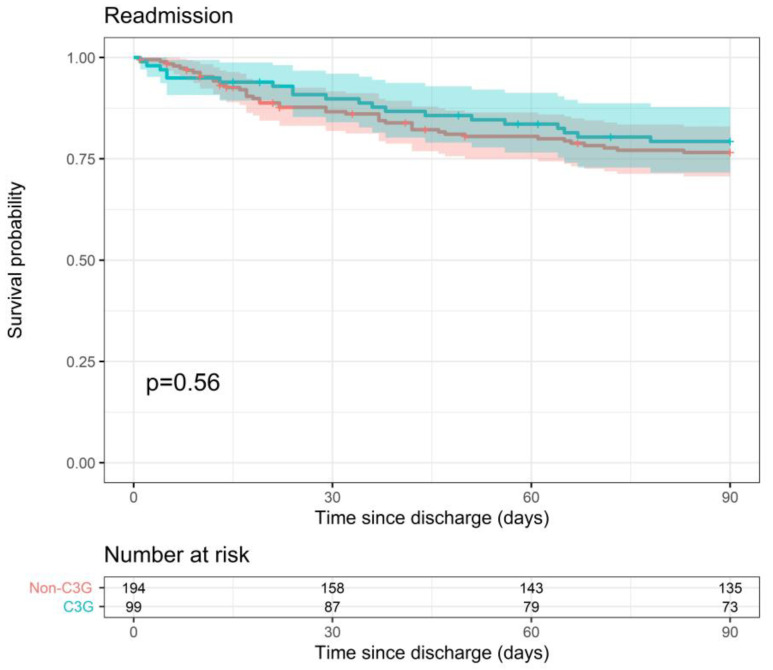
Probability of readmission within 90 days after hospital discharge for 3GC- and non-3GC-therapy groups for patients presenting BSIs due to any AmpC-E.

Readmission within 90 days of hospital discharge showed no significant difference between the 3GC- and non-3GC-definitive-therapy groups (90-day readmission probability of 20.7% vs. 23.4%, *p* = 0.56) (Figure 5). There was no association with severity indicators and therefore no need for statistical adjustment.

Median length-of-stay was significantly higher in the 3GC-definitive-therapy group (38 days vs. 21 days, *p* = 0.0003), as was in-hospital mortality (23.3% vs. 13.4%, *p* = 0.019). Both displayed simple associations with severity scores. To further explore this, we employed multiple models to compare the therapy groups. Even after adjusting for severity scores, significant differences between the therapy groups persisted (Supplementary Table S4).

### 3.4. Outcomes for First Subgroup Analysis—3GCs vs. Cefepime/Carbapenems for All AmpC-E

Guidelines generally recommend cefepime and carbapenems for serious infections due to AmpC-E [9]. In light of this, we performed a subgroup analysis comparing the same primary and secondary outcomes for patients treated with 3GCs (n = 129) vs. patients treated with the current standard-of-care, namely cefepime or meropenem (n = 37). Meropenem is currently the only carbapenem available in Belgium.

The primary outcome of the OTF was similar between both therapy groups (46.5% vs. 48.6%, *p* = 0.81). The logistic regression model showed similar odds of the OTF with the 3GC definitive therapy compared to the cefepime/meropenem definitive therapy (OR 0.91, 95%CI 0.44–1.91). There was no statistically significant difference between therapy groups regarding 90-day all-cause mortality (survival probability of 72.9% vs. 70.3%, *p* = 0.92), 90-day reinfection (9.1% vs. 8.8%, *p* = 0.96), 90-day readmission (20.7% vs. 18.5%, *p* = 0.82), and in-hospital mortality (23.3% vs. 24.3%, *p* = 0.89). Median length-of-stay was significantly lower in the 3GC-definitive-therapy group (38 days vs. 52 days, *p* = 0.024) (Supplementary Tables S5 and S6).

### 3.5. Outcomes for Second Subgroup Analysis—3GCs vs. Non-3GCs for High-Risk AmpC-E

According to recent studies, some AmpC-E exhibit a greater propensity to express AmpC at high levels when exposed to inducers [6]. These include the *E. cloacae* complex, *K. aerogenes*, and *C. freundii*. Consequently, we conducted a subgroup analysis focused on patients presenting BSIs due to these specific pathogens. While *H. alvei* is also categorized as a high-risk AmpC-E, it was omitted due to its relevance to only a single patient within our cohort. This subgroup analysis compares the same primary and secondary outcomes between the 3GC definitive therapy (n = 65) and non-3GC definitive therapy (n = 141).

The primary outcome of the OTF was comparable between therapy groups (43.1% vs. 36.9%, *p* = 0.37). The simple logistic regression model showed similar odds of the OTF with the 3GC definitive therapy compared to non-3GC definitive therapy (OR 1.30, 95%CI 0.71–2.36). There was no statistically significant difference between groups regarding 90-day all-cause mortality (survival probability of 78.7% vs. 78.5%, *p* = 0.77), 90-day readmission (20.0% vs. 24.7%, *p* = 0.38), and in-hospital mortality (20.0% vs. 9.9%, *p* = 0.051). Reinfection within 90 days was higher in the non-3GC-therapy group (1.6% vs. 9.2%, *p* = 0.028). Median length-of-stay was higher in the 3GC-therapy group (30 days vs. 20 days, *p* = 0.001) (Supplementary Tables S7 and S8).

## 4. Discussion

Reluctance to treat AmpC-E infections with 3GCs stems from two landmark studies conducted by Chow et al. in 1991 (prospective, 129 patients presenting *Enterobacter* spp. BSI) and Kaye et al. in 2001 (retrospective, 477 patients with *Enterobacter* spp. all-site infections). Both reported a 3GC-resistance-emergence rate of 19% (6/31 and 31/161, respectively) [10,11]. Jacobson et al. and Schwaber et al. similarly concluded that 3GC and piperacillin therapies were associated with a higher risk for 3GC-resistance development in AmpC-E [12,20]. However, recent prospective and retrospective studies indicate relatively low rates of 3GC-resistance emergence, ranging from 1.9 to 5% [12,13,14,15].

Furthermore, the development of microbiological resistance may not necessarily lead to clinical failure. In a retrospective study by Derrick et al. in 2020, which included 381 patients with *Enterobacter* spp., *Citrobacter* spp., and *Serratia* spp. BSIs, no statistically significant differences were found regarding in-hospital mortality, 30-day readmission, and 90-day reinfection between patients treated with 3GC and non-3GC definitive therapies [16]. Similarly, a 2021 retrospective analysis by Drozdinsky et al., focusing on 277 *Enterobacter* spp. BSIs, found no significant advantage in using carbapenems over alternative antibiotics, including 3GCs [17]. Another prospective study by Mounier et al. between 2017 and 2020, involving 177 ICU patients with AmpC-E all-site infections, actually found that cefotaxime was associated with a protective effect against clinical failure (defined as death or the need to switch to broader-spectrum antibiotics) [18].

Both CLSI and EUCAST guidelines recommend reporting AmpC-E susceptibility, with a footnote suggesting the possibility of 3GC-resistance development during therapy [21,22]. The Infectious Diseases Society of America (IDSA) advises against 3GC therapy for the *E. cloacae* complex, *K. aerogenes*, and *C. freundii*, and allows 3GC therapy if susceptible for *S. marcescens*, *P. stuartii*, and *M. morganii*, and does not take a definitive stance on *H. alvei* due to limited research [9]. This species-specific approach is based on two studies focused on microbiological outcomes. In a 2021 in vitro analysis, Kohlmann et al. identified the *E. cloacae* complex, *K. aerogenes*, *C. freundii*, and *H. alvei* as presenting higher mutation rates than other AmpC-E [6]. In 2008, Choi et al. prospectively studied 732 AmpC-E all-site infections and found a resistance-emergence rate during 3GC therapy of 5% (11/218), with eight *E. cloacae* complexes, two *K. aerogenes*, and one *C. freundii*. However, the small number of patients in whom resistance emerged did not allow for conclusive evidence regarding clinical outcomes [14].

If 3GCs are to be avoided, IDSA guidelines recommend primarily 4GCs (such as cefepime) and carbapenems [9]. Piperacillin–tazobactam therapy is discouraged by the IDSA due to the limited ability of tazobactam to inhibit AmpC hydrolysis in vitro [5,23]. A recent meta-analysis of 11 studies and the MERINO-2 randomized controlled trial have compared the β-lactam/β-lactamase inhibitor vs. carbapenem therapy in AmpC-E infections and found no difference in mortality. However, both studies have limitations that prevent definitive conclusions about the noninferiority of piperacillin–tazobactam [24,25]. Non-β-lactam options, such as TMP-SMX and fluoroquinolones, should only be considered after demonstrating antibiotic susceptibility [9].

Both cefepime and carbapenems present disadvantages. Cefepime has broader ecological consequences than 3GCs according to the classification established by Weiss et al. [26], and can cause neurotoxicity, especially in patients with decreased kidney function [27]. The prevalence of carbapenem resistance in Gram-negative bacteria is increasing in many countries, and efforts should be made to limit carbapenem use to slow its emergence [28]. Additionally, the high cost of carbapenems is a concern, especially in countries with primarily out-of-pocket health expenditure [29].

In this retrospective study including 353 patients with 3GC-susceptible AmpC-E BSI, the primary outcome of the OTF was comparable between the 3GC- and non-3GC-therapy groups. The components of the OTF (90-day all-cause mortality, 90-day reinfection, and 90-day readmission) were also similar. The noninferiority of 3GC definitive therapy in our findings concurs with the recent literature studying clinical outcomes with a more extended analysis period for mortality and readmission (90 days vs. 30 days) [16,17]. In-hospital all-cause mortality was significantly higher in the 3GC-therapy group in our study, which may be linked to a higher illness severity and a longer length of stay compared to the non-3GC-therapy group. The disparity in the length of stay could be attributed to two key factors. Firstly, the higher severity of illness in the 3GC-therapy group potentially contributed to increased complications, subsequently prolonging the hospitalization period. Secondly, the availability of oral antibiotic options, such as fluoroquinolones and TMP-SMX, facilitated the early discharge in the non-3GC cohort. Notably, the absence of oral 3GC formulations in Belgium meant that patients in the 3GC group had to remain hospitalized for the entire antibiotic course.

One limitation within our principal study involves the aggregation of all “non-3GC” agents into a single category. As previously indicated, current guidelines advocate for cefepime or carbapenems as the standard-of-care for AmpC-E infections. To decisively validate 3GCs as a therapeutic choice, an ideal study design would compare 3GCs to the established standard-of-care. To address this, we conducted a subgroup analysis with analogous outcomes for BSIs due to any AmpC-E, but focused on 3GC therapy vs. cefepime or meropenem therapy. We discovered no statistically significant disparities between therapy groups in terms of the OTF, all-cause 90-day mortality, 90-day reinfection, 90-day readmission, and in-hospital all-cause mortality. The sole difference concerned the median length-of-stay in favor of the 3GC-therapy group. These conclusions are limited by the small sample size of our cefepime/meropenem cohort (n = 37). Nonetheless, these findings appear to support the role of 3GCs as a credible alternative to the existing standard-of-care.

Organisms presenting high potential for AmpC expression (i.e., the *E. cloacae* complex, *K. aerogenes*, *C. freundii*, and *H. alvei*) [6] are slightly under-represented in our study (58.6%) compared to previous articles (64.3–100%) [16,17]. This relatively high proportion of organisms less likely to produce AmpC, such as *S. marcescens*, *M. morganii*, and *P. stuartii*, might have influenced clinicians towards 3GC use. This could explain the more balanced proportions of the 3GC- vs. non-3GC-therapy groups (37% vs. 63%, respectively) compared to Derrick et al. (17% vs. 87%) and Drozdinsky et al. (26% vs. 74%) [16,17]. In order to enhance the precision of our conclusions, we performed a subgroup analysis with similar treatments (3GCs vs. non-3GCs) and similar outcomes, focusing on patients presenting with high-risk AmpC-E BSI. We found no statistically significant difference in the OTF, 90-day all-cause mortality, 90-day readmission, and in-hospital all-cause mortality. The only differences concerned 90-day reinfection in favor of the 3GC-therapy group and the median length-of-stay in favor of the non-3GC-therapy group. These findings bolster the viability of 3GCs as a therapeutic option for BSIs, even among patients presenting with high-risk AmpC-E.

Our study has several limitations, first and foremost the retrospective and monocentric design. The use of a composite outcome may lead to misinterpretation, although this limitation was mitigated by the absence of significant differences between therapy groups for the OTF, as well as each of its individual components. Although comparable to previous studies, the sample size is still limited [16,17]. The 3GC group had significantly higher illness severity. In our principal analysis, we studied outcomes for all AmpC-E BSIs without evaluating each organism individually, and the subgroup analysis regarding high-risk AmpC-E was constrained by a smaller sample size. As mentioned, alternatives to 3GCs were grouped as “non-3GC” in the principal analysis, and the subgroup analysis comparing 3GCs vs. standard-of-care also encountered limitations due to a reduced sample size. Limitations within the medical record software hindered the extraction of information regarding source controls, such as catheter removal and abscess puncture or excision, as well as the duration and dosing of antibiotic therapies. We did not assess potential adverse effects associated with each antibiotic therapy, including the occurrence of secondary infections, whether fungal or bacterial (including those related to Clostridium difficile).

The primary strength of our study lies in the thorough review of all medical files from the index blood culture to 90 days post-discharge. The collected information was therefore more precise and contextualized compared to digitally extracted data based on arbitrary criteria. The availability of all commonly used antibiotics with Gram-negative coverage, including cefepime, during the study period, was confirmed by the hospital pharmacy. Therefore, antibiotic availability did not influence therapeutic decisions.

Ideally, a randomized controlled trial comparing 3GCs to cefepime or carbapenems for the treatment of high-risk AmpC-E BSIs would provide the most robust evidence. However, highly bioavailable oral options, such as fluoroquinolones or TMP-SMX, are very attractive to patients and would make enrollment difficult.

## 5. Conclusions

Our study demonstrates similar risk of the OTF between 3GC and non-3GC definitive therapies for AmpC-E BSIs, with comparable 90-day mortality, 90-day reinfection, and 90-day readmission. Both subgroup analyses, one involving high-risk AmpC-E and the other comparing 3GCs against the current standard-of-care, consistently support the notion that 3GCs can provide a valid therapeutic alternative to cefepime and carbapenems. These findings contradict current guidelines and highlight the need for further investigation on the topic, as we eagerly seek broad-spectrum β-lactams and carbapenem-sparing strategies.

## Figures and Tables

**Table 1:** Comparison of patients’ characteristics between therapy groups.

Variable	3GC Definitive Therapy *n* = 129	Non-3GC Definitive Therapy *n* = 224	*p*-Value
General characteristics			
Sex, male ^1^	86 (66.7)	164 (73.2)	0.19
Age (years) ^2^	66.2 ± 14.4	64.3 ± 14.6	0.23
Weight (kg) ^2^	78.6 ± 18.4	76.8 ± 18.7	0.42
Height (m) ^2^	169.6 ± 10.0	171.1 ± 8.7	0.23
BMI (kg/m^2^) ^2^	27.0 ± 6.7	26.0 ± 6.0	0.24
Renal function (mL/min) ^3^	65.1 (34.9;113.0)	74.6 (50.9;106.6)	0.14
Penicillin allergy ^1^	12 (9.3)	20 (8.9)	0.91
Charlson comorbidity index ^2^	4.2 ± 2.9	4.1 ± 3.0	0.70
Antimicrobial therapy ^1^			
Adequate empiric therapy	120 (93.0)	209 (93.3)	0.92
Adequate definitive therapy	128 (99.2)	222 (99.1)	1.00 ^4^
Aminoglycoside use	25 (19.4)	29 (12.9)	0.11
Severity of illness			
ICU admission ^1^	54 (41.9)	57 (25.4)	**0.0014**
qPitt score ^3^	1.50 (1.00;2.00)	1.00 (0.00;2.00)	**0.0017**
qSOFA score ^3^	1.00 (0.00;2.00)	1.00 (0.00;2.00)	**0.0017**
Sedated and intubated ^1^	19 (14.7)	22 (9.8)	0.17
Source ^1^			0.014
Respiratory	36 (27.9)	46 (20.5)	
Urinary tract	17 (13.2)	51 (22.8)	
Catheter-related	28 (21.7)	38 (17.0)	
Intra-abdominal	15 (11.6)	42 (18.8)	
Soft tissue	11 (8.5)	10 (4.5)	
Other	8 (6.2)	5 (2.2)	
Unknown	14 (10.9)	32 (14.3)	
Pathogen ^1^			0.21 ^4^
*E. cloacae* complex	44 (34.1)	103 (46.0)	
*S. marcescens*	44 (34.1)	55 (24.6)	
*M. morganii*	19 (14.7)	25 (11.2)	
*K. aerogenes*	10 (7.7)	23 (10.3)	
*C. freundii*	11 (8.5)	15 (6.7)	
*P. stuartii*	1 (0.8)	2 (0.9)	
*H. alvei*	0 (0.0)	1 (0.5)	

^1^ Data reported as n (%); ^2^ Data reported as mean ± SD; ^3^ Data reported as median (Q1;Q3) and nonparametric Kruskal–Wallis test; ^4^ Exact Fisher test.

**Table 2:** Comparison of empiric and definitive therapy between groups.

	Empiric Therapy		Definitive Therapy	
Antibiotic	3GC Definitive Therapy ^1^ *n* = 129	Non-3GC Definitive Therapy ^1^ *n* = 224	3GC Definitive Therapy ^1^ *n* = 129	Non-3GC Definitive Therapy ^1^ *n* = 224
Penicillins				
Amoxicillin	0 (0.0)	1 (0.4)	/	0 (0.0)
Amoxicillin + clavulanate	4 (3.1)	5 (2.2)	/	1 (0.5)
Flucloxacillin	1 (0.8)	1 (0.4)	/	0 (0.0)
Temocillin	0 (0.0)	2 (0.9)	/	4 (1.8)
Piperacillin + tazobactam	32 (24.8)	103 (46.0)	/	56 (25.0)
2GC			/	
Cefuroxime	1 (0.8)	4 (1.8)	/	3 (1.3)
3GC				
Cefotaxime	33 (25.6)	5 (2.2)	57 (44.2)	/
Ceftriaxone	22 (17.1)	18 (8.0)	44 (34.1)	/
Ceftazidime	21 (16.3)	6 (2.7)	28 (21.7)	/
4GC				
Cefepime	6 (4.7)	16 (7.1)	/	18 (8.0)
Carbapenems			/	
Meropenem	7 (5.4)	24 (10.7)	/	19 (8.5)
Fluoroquinolones				
Ciprofloxacin	0 (0.0)	25 (11.2)	/	84 (37.5)
Moxifloxacin	0 (0.0)	4 (1.8)	/	3 (1.3)
Others				
Minocycline	0 (0.0)	1 (0.4)	/	1 (0.5)
Tigecycline	1 (0.8)	1 (0.4)	/	0 (0.0)
TMP-SMX	1 (0.8)	8 (3.6)	/	35 (15.6)

^1^ Data reported as n (%).

**Table 3:** Comparison of clinical outcomes between 3GC- and non-3GC-therapy groups for patients presenting BSIs due to any AmpC-E.

Outcome	3GC Definitive Therapy *n* = 129	Non-3GC Definitive Therapy *n* = 224	*p*-Value ^1^
OTF ^2^	60 (46.5)	93 (41.5)	0.36
Survival for:			
Death (all-cause) within 90 days ^3^	72.9	75	0.51 ^a^
Reinfection within 90 days ^3^	90.9	95.6	0.10 ^b^
Readmission within 90 days ^3^	79.3	76.6	0.56 ^c^
Length of stay ^4^ (days)	38.0 (15.0;61.0)	21.0 (10.0;43.5)	0.0003
In-hospital all-cause mortality ^2^	30 (23.3)	30 (13.4)	0.019 ^a^

^1^ Simple analysis results; ^2^ Data reported as n (%) and tested using logistic regression; ^3^ Data reported as 90-day survival probability (%) and tested using a simple Cox-regression model; ^4^ Data reported as median (Q1;Q3) and nonparametric Kruskal–Wallis test; ^a^ Significant association with qPitt score in multiple analysis, and the adjusted model did not alter the conclusion; ^b^ Slight association with ICU admission in multiple analysis, and the adjusted model did not alter the conclusion; ^c^ No association with severity scores in multiple analysis.

## Data Availability

The data presented in this study are openly available in Figshare, under the DOI 10.6084/m9.figshare.24115467.

## Supplementary Materials

The following supporting information can be downloaded at: https://www.mdpi.com/article/10.3390/pathogens12091152/s1, Supplementary Table S1—Comparison of comorbidities between ICD-9 and ICD-10; Supplementary Table S2—Comparison of components of Charlson comorbidity index between therapy groups; Supplementary Table S3—Comparison of events involved in qSOFA and qPitt scores between therapy groups; Supplementary Table S4—Simple and adjusted models with regard to association to severity scores; Supplementary Table S5—Comparison of clinical outcomes between 3GC and cefepime/meropenem therapy groups for patients presenting BSIs due to any AmpC-E; Supplementary Table S6—Simple (HR/OR) and adjusted (aHR/aOR) estimates for secondary outcomes in regards to the 1st subgroup analysis—3GCs vs. cefepime/meropenem for patients presenting BSIs due to any AmpC-E; Supplementary Table S7—Comparison of clinical outcomes between 3GC- and non-3GC-therapy groups for patients presenting BSIs due to *E. cloacae* complex, *K. aerogenes*, and *C. freundii;* Supplementary Table S8—Simple (HR/OR) and adjusted (aHR/aOR) estimates for secondary outcomes in regards to the 2nd subgroup analysis—3GCs vs. non-3GCs for patients presenting BSIs due to the *E. cloacae* complex, *K. aerogenes*, and *C. freundii*.
