# Supplementary material for: Clinical Outcomes of Third-Generation Cephalosporin Definitive Therapy for Bloodstream Infections Due to Enterobacterales with Potential AmpC Induction: A Single-Center Retrospective Study
Source: Pathogens. 2023 Sep 11;12(9):1152. doi: 10.3390/pathogens12091152 (PMC10537439; doi:10.3390/pathogens12091152)
Supplement: Supplementary file 1 [file pathogens-12-01152-s001.zip › pathogens-2533088-supplementary.pdf]

pathogens MDPI

Supplementary Table S1 - Comparison of comorbidities between ICD-9 and ICD-10

| Item | Score | ICD-9[1] N=86 | ICD-10[1] N=267 | p-value |
|---|---|---|---|---|
| Myocardial infarction | 1 | 10 (11.6) | 49 (18.4) | 0.15 |
| Congestive heart failure | 1 | 26 (30.2) | 62 (23.2) | 0.19 |
| Peripheral vascular disease | 1 | 16 (18.6) | 59 (22.1) | 0.49 |
| Cerebrovascular disease | 1 | 14 (16.3) | 62 (23.2) | 0.17 |
| Dementia | 1 | 3 (3.5) | 13 (4.9) | 0.77[3] |
| Chronic pulmonary disease | 1 | 32 (37.2) | 82 (30.7) | 0.26 |
| Rheumatic disease | 1 | 2 (2.3) | 9 (3.4) | 1.00[3] |
| Peptic ulcer disease | 1 | 7 (8.1) | 25 (9.4) | 0.73 |
| Mild liver disease | 1 | 12 (14.0) | 35 (13.1) | 0.84 |
| Diabetes without chronic complication | 1 | 16 (18.6) | 50 (18.7) | 0.98 |
| Diabetes with chronic complication | 2 | 5 (5.8) | 31 (11.6) | 0.12 |
| Hemiplegia or paraplegia | 2 | 10 (11.6) | 42 (15.7) | 0.35 |
| Renal disease | 2 | 20 (23.3) | 75 (28.1) | 0.38 |
| Any malignancy[2] | 2 | 26 (30.2) | 87 (32.6) | 0.68 |
| Moderate or severe liver disease | 3 | 3 (3.5) | 22 (8.2) | 0.14 |
| Metastatic solid tumor | 3 | 18 (20.9) | 47 (17.6) | 0.49 |
| AIDS/HIV | 6 | 0 (0.0) | 2 (0.7) | 1.00[3] |

[1] Data reported as n (%)
[2] Including lymphoma and leukemia, except malignant neoplasm of skin
[3] Exact Fisher test

Supplementary Table S2 – Comparison of components of Charlson comorbidity index between therapy groups.

| Item | Score | 3GC definitive therapy[1] N=129 | Non-3GC definitive therapy[1] N=224 | p-value[2] |
|---|---|---|---|---|
| Myocardial infarction | 1 | 19 (14.7) | 40 (17.9) | 0.45 |
| Congestive heart failure | 1 | 41 (31.8) | 47 (21.0) | **0.024** |
| Peripheral vascular disease | 1 | 29 (22.5) | 46 (20.5) | 0.67 |
| Cerebrovascular disease | 1 | 31 (24.0) | 45 (20.1) | 0.39 |
| Dementia | 1 | 7 (5.4) | 9 (4.0) | 0.54 |
| Chronic pulmonary disease | 1 | 47 (36.4) | 67 (29.9) | 0.21 |
| Rheumatic disease | 1 | 3 (2.3) | 8 (3.6) | 0.75[3] |
| Peptic ulcer disease | 1 | 10 (7.8) | 22 (9.8) | 0.51 |
| Mild liver disease | 1 | 17 (13.2) | 30 (13.4) | 0.95 |
| Diabetes without chronic complication | 1 | 23 (17.8) | 43 (19.2) | 0.75 |
| Diabetes with chronic complication | 2 | 15 (11.6) | 21 (9.4) | 0.50 |
| Hemiplegia or paraplegia | 2 | 20 (15.5) | 32 (14.3) | 0.76 |
| Renal disease | 2 | 43 (33.3) | 52 (23.2) | **0.039** |
| Any malignancy[3] | 2 | 38 (29.5) | 75 (33.5) | 0.44 |
| Moderate or severe liver disease | 3 | 8 (6.2) | 17 (7.6) | 0.62 |
| Metastatic solid tumor | 3 | 18 (14.0) | 47 (21.0) | 0.10 |
| AIDS/HIV | 6 | 1 (0.8) | 1 (0.4) | 1.00[4] |

[1] Data reported as n (%)
[2] In order to avoid the problem of multiple testing, p-values are compared to Bonferroni adjusted significant level 0.05/17 = 0.0029
[3] Including lymphoma and leukemia, except malignant neoplasm of skin
[4] Exact Fisher test

Supplementary Table S3 – Comparison of events involved in qSOFA and qPitt scores between therapy groups

| **Item** | **3GC definitive therapy[1] N=129** | **Non-3GC definitive therapy[1] N=224** | **p-value[2]** |
|---|---|---|---|
| RR > 22/min | 31 (28.2) | 27 (13.4) | **0.0013** |
| RR > 25/min | 31 (28.2) | 26 (12.9) | **0.0008** |
| Altered mental status | 44 (40.0) | 42 (20.8) | **0.0003** |
| Cardiac arrest | 4 (3.1) | 7 (3.1) | 1.00[3] |
| Systolic BP < 100 mmHg | 72 (55.8) | 122 (54.5) | 0.81 |
| Systolic BP < 90 mmHg | 52 (40.3) | 79 (35.3) | 0.35 |
| Temperature < 36°C | 55 (42.6) | 99 (44.2) | 0.78 |

[1] Data reported as n (%)
[2] In order to avoid the problem of multiple testing, p-values are compared to Bonferroni adjusted significant level 0.05/7 = 0.0071
[3] Exact Fisher test

Supplementary Table S4 – Simple (HR/OR) and adjusted (aHR/aOR) estimates for secondary outcomes in regards to the principal analysis – 3GCs vs. non-3GCs for patients presenting BSIs due to any AmpC-E

| **Outcomes** | **Death (all-cause) within 90 days[1]** | | **Reinfection within 90 days[1]** | | **Readmission within 90 days[1]** | | **In-hospital mortality[2]** | |
|---|---|---|---|---|---|---|---|---|
| **Covariates** | **HR (CI95%)** | **aHR (CI95%)** | **HR (CI95%)** | **aHR (CI95%)** | **HR (CI95%)** | **aHR (CI95%)** | **OR (CI95%)** | **aOR (CI95%)** |
| 3CG vs. non-3CG therapy | 1.15 (0.76-1.76) | 0.97 (0.60-1.55) | 2.10 (0.87-5.25) | 1.85 (0.74-4.60) | 0.87 (0.50-1.47) | - | 1.96 (1.12-3.44) | 1.58 (2.80-3.11) |
| qPitt | 1.54 (1.28-1.84)* | 1.54 (1.28-1.85)* | 1.07 (0.64-1.80) | - | 0.98 (0.76-1.25) | - | 2.25 (1.70-3.06) | 2.20 (1.65-2.98)* |
| qSOFA | 1.78 (1.40-2.28)* | - | 1.60 (0.86-3.00) | - | 0.99 (0.73-1.33) | - | 2.84 (1.97-4.20) | - |
| ICU admission | 2.28 (1.50-3.45)* | - | 2.55 (1.03-6.27)* | 2.27 (0.91-5.68) | 0.54 (0.28-1.10) | - | 5.70 (3.18-10.47) | - |

[1] Cox regression model applied
[2] Logistic regression model applied
* 95% level of significance
HR - Hazard Ratio ; aHR - adjusted Hazard Ratio ; OR-Odds Ratio ; aOR - adjusted Odds Ratio ; CI95%-95% Confidence Intervals

Supplementary Table S5 – Comparison of clinical outcomes between 3GC and cefepime/meropenem therapy groups for patients presenting BSIs due to any AmpC-E

| **Outcome** | **3GC definitive therapy N=129** | **Cefepime/meropenem definitive therapy N=37** | **p-value[1]** |
|---|---|---|---|
| OTF[2] | 60 (46.5) | 18 (48.6) | 0.81 |
| Survival for : | | | |
| Death (all-cause) within 90 days[3] | 72.9 | 70.3 | 0.92[a] |
| Reinfection within 90 days[3] | 90.9 | 91.2 | 0.96[b] |
| Readmission within 90 days[3] | 79.3 | 81.5 | 0.82[b] |
| Length of stay[4] (days) | 38.0 (15.0;61.0) | 52.0 (28.0;85.0) | 0.024 |
| In-hospital all-cause mortality[2] | 30 (23.3) | 9 (24.3) | 0.89[a] |

[1] Simple analysis results
[2] Data reported as n (%) and tested using logistic regression
[3] Data reported as 90-day survival probability (%) and tested using a simple Cox-regression model
[4] Data reported as median (Q1;Q3) and non-parametric Kruskal-Wallis test
[a] Significant association with qPitt score in multiple analysis, the adjusted model did not alter the conclusion
[b] No association with severity scores in multiple analysis

Supplementary Table S6 – Simple (HR/OR) and adjusted (aHR/aOR) estimates for secondary outcomes in regards to the 1st subgroup analysis – 3GCs vs. cefepime/meropenem for patients presenting BSIs due to any AmpC-E

| **Outcomes** | **Death (all-cause) within 90 days[1]** | | **Reinfection within 90 days[1]** | | **Readmission within 90 days[1]** | | **In-hospital mortality[2]** | |
|---|---|---|---|---|---|---|---|---|
| **Covariates** | **HR (CI95%)** | **aHR (CI95%)** | **HR (CI95%)** | **aHR (CI95%)** | **HR (CI95%)** | **aHR (CI95%)** | **OR (CI95%)** | **aOR (CI95%)** |
| 3CG vs. non-3CG therapy | 0.97 (0.50-1.90) | 0.92 (0.44-1.91) | 1.03 (0.28-3.73) | 2.30 (0.28-19.02) | 1.12 (0.42-2.97) | 1.03 (0.39-2.78) | 0.94 (0.41-2.31) | 0.83 (0.32-2.33) |
| qPitt | 1.46 (1.13-1.88)* | 1.46 (1.13-1.89)* | 0.93 (0.500-1.73) | 1.35 (0.64-2.86) | 1.04 (0.73-1.47) | - | 1.84 (1.30-2.70)* | 1.83 (1.29-2.69)* |
| qSOFA | 1.53 (1.08-2.16)* | - | 1.28 (0.60-2.72) | - | 0.91 (0.58-1.43) | - | 2.08 (1.34-3.35)* | - |
| ICU admission | 1.91 (1.07-3.42)* | - | 1.84 (0.62-5.45) | - | 0.60 (0.25-1.45) | 0.61 (0.25-1.47) | 2.93 (1.41-6.33)* | - |

[1] Cox regression model applied
[2] Logistic regression model applied
* 95% level of significance
HR - Hazard Ratio ; aHR - adjusted Hazard Ratio ; OR-Odds Ratio ; aOR - adjusted Odds Ratio ; CI95%-95% Confidence Intervals

Supplementary Table S7 – Comparison of clinical outcomes between 3GC and non-3GC therapy groups for patients presenting BSIs due to the *E. cloacae* complex, *K. aerogenes* and *C. freundii*.

| **Outcome** | **3GC definitive therapy N=65** | **Non-3GC definitive therapy N=141** | **p-value[4]** |
|---|---|---|---|
| OTF[3] | 28 (43.1) | 52 (36.9) | 0.37 |
| Survival for : | | | |
| Death (all-cause) within 90 days[1] | 78.7 | 78.5 | 0.77[a] |
| Reinfection within 90 days[1] | 98.4 | 90.8 | 0.028[b] |
| Readmission within 90 days[1] | 80.0 | 75.3 | 0.38[a] |
| Length of stay[2] (days) | 30.0 (13.0;70.0) | 20.0 (11.0;35.0) | 0.001 |
| In-hospital all-cause mortality[3] | 13 (20.0) | 14 (9.9) | 0.051[a] |

[1] Data reported as 90-day survival probability (%) and tested using a simple Cox-regression model
[2] Data reported as median (Q1;Q3) and non-parametric Kruskal-Wallis test
[3] Data reported as n (%) and tested using logistic regression
[4] Simple analysis results
[a] Significant association with qSOFA score in multiple analysis, the adjusted model did not alter the conclusion
[b] No association with severity scores in multiple analysis

Supplementary Table S8 – Simple (HR/OR) and adjusted (aHR/aOR) estimates for secondary outcomes in regards to the 2ndsubgroup analysis – 3GCs vs. non-3GCs for patients presenting BSIs due to the *E. cloacae* complex, *K. aerogenes* and *C. freundii*.

| **Outcomes** | **Death (all-cause) within 90 days[1]** | | **Reinfection within 90 days[1]** | | **Readmission within 90 days[1]** | | **In-hospital mortality[2]** | |
|---|---|---|---|---|---|---|---|---|
| **Covariates** | **HR (CI95%)** | **aHR (CI95%)** | **HR (CI95%)** | **aHR (CI95%)** | **HR (CI95%)** | **aHR (CI95%)** | **OR (CI95%)** | **aOR (CI95%)** |
| 3CG vs. non-3CG therapy | 1.10 (0.58-2.07) | 0.75 (0.35-1.62) | 6.28 (1.22-32.41)* | 3.32 (0.54-20.39) | 0.73 (0.36-1.49) | 0.77 (0.36-1.64) | 2.27 (0.98-5.18) | 1.66 (0.58-4.62) |
| qPitt | 1.28 (0.97-1.68) | - | 1.17 (0.55-2.45) | - | 1.09 (0.83-1.42) | - | 1.81 (1.20-2.80)* | - |
| qSOFA | 1.62 (1.11-2.36)* | 1.65 (1.13-2.42)* | 1.97 (0.73-5.34) | 1.78 (0.61-5.19) | 1.22 (0.85-1.76) | 1.53 (1.02-2.77)* | 2.50 (1.40-4.66)* | 2.40 (1.32-4.52)* |
| ICU admission | 1.82 (0.99-3.35) | - | 1.23 (0.24-6.33) | - | 0.52 (0.22-1.23) | 0.33 (0.11-1.02) | 4.60 (2.00-10.92)* | - |

[1] Cox regression model applied
[2] Logistic regression model applied
* 95% level of significance
HR - Hazard Ratio ; aHR - adjusted Hazard Ratio ; OR-Odds Ratio ; aOR - adjusted Odds Ratio ; CI95%-95% Confidence Intervals
